# Acid Suppression in Mild‐Moderate Laryngomalacia Without GERD: A Randomized Controlled Trial

**DOI:** 10.1002/lary.32471

**Published:** 2025-08-05

**Authors:** Amber D. Shaffer, Zainab Balogun, Allison B. J. Tobey, Raymond C. Maguire, Jeffrey P. Simons, Joseph E. Dohar, Jennifer L. Mccoy, Marina V. Rushchak, Reema Padia

**Affiliations:** ^1^ Department of Otolaryngology‐Head and Neck Surgery University of Pittsburgh Medical Center Pittsburgh Pennsylvania USA

**Keywords:** famotidine, infant, laryngomalacia, stridor

## Abstract

**Objective:**

To determine the efficacy of acid suppression therapy (AST) for the treatment of gastroesophageal reflux (GER) and airway symptoms in infants with mild to moderate laryngomalacia.

**Methods:**

From 2020 to 2023, infants ≤ 6 months old with laryngomalacia at a tertiary children's hospital were randomized to famotidine and feeding modifications (AST) or feeding modifications alone (no‐AST). Laryngomalacia Airway Symptom Score (LASS) and Infant Gastroesophageal Reflux Questionnaire (I‐GERQ‐R) were completed by guardians. Of 343 patients approached, 257 were excluded due to severe laryngomalacia on LASS, severe GER (I‐GERQ‐*R* ≥ 16), prior AST, no laryngomalacia on laryngoscopy, recommendation for supraglottoplasty, and/or other airway anomalies. Twenty‐one declined participation. LASS and I‐GERQ‐R were again completed 1–6 months following enrollment.

**Results:**

Sixty‐five patients enrolled; 40/65 (62%) followed up at a mean of 3.1 months (SD 1.4 months). Of these 40, 10 (25%) had mild and 30 (75%) had moderate laryngomalacia. Median I‐GERQ‐R was 11 (range 5–15) at the initial appointment and 7.5 (range 0–26) at follow‐up (*p* = 0.002). Laryngomalacia resolved in 13/40 (33%) at follow‐up based on LASS (*p* < 0.001). Patients randomized to AST (*n* = 20) and no‐AST (*n* = 20) had comparable improvement on LASS (*p* = 0.3) and I‐GERQ‐R (*p* = 0.8). Additionally, the severity of laryngomalacia at initial consult did not have a significant impact on LASS (*p* = 0.3) or I‐GERQ‐R (*p* = 0.8) improvement. LASS (*ρ* = 0.423, *p* = 0.007) but not I‐GERQ‐R (*ρ* = 0.122, *p* = 0.5) improved more with longer time from consult to follow‐up.

**Conclusion:**

This small randomized controlled trial was unable to demonstrate additional benefit from AST compared to feeding modifications alone based on airway and reflux symptoms.

**Level of Evidence:**

2.

**Trial Registration:**

https://clinicaltrials.gov/study/NCT04614974

## Graphical abstract

Infants ≤ 6 months old with mild to moderate laryngomalacia at a tertiary children's hospital were randomized to famotidine and feeding modifications (AST) or feeding modifications alone (no‐AST). Patients randomized to AST (*n* = 20) and no‐AST (*n* = 20) had comparable improvement on Laryngomalacia Airway Symptom Score and Infant Gastroesophageal Reflux Questionnaires. This small randomized controlled trial was unable to demonstrate additional benefit from AST compared to feeding modifications alone based on airway and reflux symptoms.

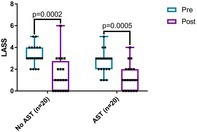

## 1 Introduction

Laryngomalacia accounts for 45%–75% of all congenital stridor [1]. About 20% of patients with laryngomalacia present with life‐threatening symptoms such as severe airway obstruction and feeding difficulties that require invasive interventions [2]. The most common presentation of laryngomalacia is inspiratory stridor from supraglottic collapse during the inspiratory phase of respiration, producing the classic harsh, squeaky sound [3]. Patients can also present with symptoms of gastroesophageal reflux (GER) such as regurgitation, cough, choking, slow feeding, and emesis. Other less common symptoms include suprasternal retractions, cyanosis, pectus excavatum, tachypnea, and obstructive sleep apnea [3].

GER affects about 65%–100% of infants with laryngomalacia, which can result in weight loss and failure to thrive [3, 4]. Thompson et al. suggested in 2010 that infants with laryngomalacia symptomatic for GER be treated with acid suppression therapy (AST) [5], which was reiterated in International Pediatric ORL Group consensus recommendations [6]. Primary treatment options for laryngomalacia are observation, with reflux precautions when GER is present, or surgical intervention with supraglottoplasty (SGP) in severe cases. While AST remains an empiric intervention for patients with laryngomalacia, high‐quality evidence supporting AST's efficacy in this population is limited. For example, Dang et al. sought to determine if AST prescribed for laryngomalacia was associated with improvement in symptoms, weight gain, or reduced likelihood of surgery. All patients were prescribed AST, and while weight percentile and airway and dysphagia symptoms improved at follow‐up, improvement did not differ between groups with mild/moderate versus severe laryngomalacia based on symptoms [7]. The issue is particularly relevant because symptoms of laryngomalacia resolve in 90% of cases by 18–20 months of age without operative intervention [8].

Patients with severe laryngomalacia are often surgical candidates, and AST prescription is indicated in patients with significant GER symptoms. However, the role of AST in the treatment of mild/moderate laryngomalacia in the absence of substantial GER is less clear. The present trial sought to clarify the role of AST. The hypothesis was that, in infants < 6 months old with mild/moderate laryngomalacia, AST with feeding modifications would not provide superior GER and airway symptom outcomes 1–6 months after initiation as compared to observation and feeding modifications alone.

## 2 Materials and Methods

### 2.1 Human Subjects

Following University of Pittsburgh Institutional Review Board approval (STUDY20090193), electronic medical records of all consecutive patients meeting inclusion criteria (≤ 6 months of age and presenting to airway clinic from November 2020 to May 2023 for symptoms related to noisy breathing) were screened. Exclusions were prior AST; prematurity of < 37 weeks gestational age at birth; lung disease; history of cyanosis, apnea, or failure to thrive; sleep‐induced laryngomalacia; craniofacial abnormalities or syndromes; additional airway abnormalities; or prior cardiac surgery. Patients in the airway clinic were seen by an attending physician and advanced practice provider from pediatric otolaryngology as well as a speech language pathologist.

### 2.2 Screening Procedures

The Laryngomalacia Airway Symptom Score (LASS) and the Infant Gastroesophageal Reflux Questionnaire (I‐GERQ‐R) were completed by parents/legal guardians. The novel tool LASS includes 10 questions regarding the severity of presentation extrapolated from Thompson [5] (Figure S1). While this tool has not yet undergone formal psychometric validation, it was pilot tested for face validity and clinical usability. Patients were classified as having mild laryngomalacia if responses to either questions 1 or 2 were “yes,” and all other responses were “no.” If responses to at least one question 3–5 was “yes” and all questions 6–10 were “no,” the patient was classified as having moderate laryngomalacia. Finally, if any responses to questions 6–10 were “yes,” the patient was classified as having severe laryngomalacia. Patients with severe laryngomalacia were candidates for surgical intervention and therefore were excluded from participation. In addition to these categories, the LASS was also scored 0–10 based on the number of positive (“yes”) responses.

The I‐GERQ‐R is a 12‐item instrument assessing GER symptoms. It was completed by parents/legal guardians and was scored as previously described [9]. Patients with scores ≥ 16 were considered to have true GER disease and were excluded from study participation due to AST likely being indicated.

Patients also underwent flexible laryngoscopy if presenting symptoms were consistent with laryngomalacia. Findings were recorded and defined according to Olney et al. [10] Type 1 was defined by anterior/medial collapse of supra‐arytenoid mucosa, Type 2 included short aryepiglottic folds, and Type 3 was posterior collapse of the epiglottis. These categories were not mutually exclusive. Patients were excluded from study participation if laryngoscopy was not clinically indicated, if none of these findings were observed during laryngoscopy, or if an airway anomaly in addition to laryngomalacia was observed.

### 2.3 Enrollment and Randomization

Patients passing initial chart review screening underwent randomization the day before the appointment to receive famotidine (40 mg/5 mL) and feeding modifications (AST) or feeding modifications alone (no‐AST). Feeding modifications included pacing and changes in nipple flow, formula, positioning, or thickening of feeds. A histamine‐2 receptor antagonist was selected for AST consistent with our hospital's practices for initial treatment of GER and due to concerns for increased risk of hospitalization in children under 2 years with proton pump inhibitor treatment [11]. Randomization was performed using the random number generator in Excel (Microsoft, Redmond, WA). Stratified randomization was performed based on LASS (mild or moderate). If patients met criteria based on LASS (not severe), I‐GERQ‐R (< 16), and clinical history (no exclusion criteria) at the time of the clinic visit, enrollment in the clinical trial was offered to parents/legal guardians, and written informed consent was obtained. LASS and I‐GERQ‐R were again completed 1–6 months following enrollment. If not completed at a clinic visit, the research coordinator sent the surveys via REDCap [12, 13] and/or mail.

### 2.4 Chart Review

Presenting symptoms, including noisy breathing, stridor, emesis, choking, coughing, chest wall retractions, gagging, and apnea, were recorded from the electronic medical record. Weight and percentile weight for age based on World Health Organization growth standards were also recorded at the initial visit. For participants who returned to otolaryngology within 1 year of the initial visit, symptoms at follow‐up closest to 1 year were recorded. For patients who returned to any specialty at the tertiary care center within 1 year of the initial visit, weight and weight percentile were recorded for the follow‐up closest to 1 year. Finally, operative reports were reviewed for surgical intervention (SGP).

### 2.5 Statistical Analysis

Categorical data were summarized as frequency (%). Continuous data were summarized as mean ± standard deviation (SD) if normally distributed and as median (range) if not normally distributed (Shapiro–Wilk *p* < 0.05). Baseline characteristics were compared between AST and no‐AST groups using Chi‐squared or Fisher's exact test for categorical data and t‐test (normally distributed) or Wilcoxon rank‐sum (not normally distributed) for continuous data. Primary outcome measures were change in LASS and I‐GERQ‐R scores from initial visit to follow‐up and change in airway and dysphagia symptoms assessed by chart review during the 1 year following the initial visit. The LASS and I‐GERQ‐R scores were compared between initial and follow‐up surveys using Wilcoxon signed‐rank tests. Change scores (post‐pre) were calculated for LASS and I‐GERQ‐R, and differences in the improvement in scores based on laryngomalacia severity or randomization group were evaluated using Wilcoxon rank‐sum tests. Associations between LASS or I‐GERQ‐R and time between initial and follow‐up surveys were evaluated using Spearman rank correlation. Symptoms and weight at initial and follow‐up visits were compared using McNemar's and Wilcoxon signed‐rank tests, respectively. Change in weight percentile based on laryngomalacia severity or randomization group was evaluated using Wilcoxon rank‐sum tests. Analyses were conducted using Stata/SE 16.1 (StataCorp, College Station, TX).

Based on data from Dang et al. [7], 62 participants per group were needed to address the hypothesis that there would be no difference in airway symptom improvement between AST and no‐AST groups with alpha = 0.05 and power = 0.80 (two‐tailed Fisher's exact test, G*Power 3.1) [14, 15]. Target enrollment was 160 participants (80 in the AST group and 80 in the no‐AST group, with 40 with mild laryngomalacia and 40 with moderate laryngomalacia in each group) to account for attrition.

## 3 Results

### 3.1 Enrollment

**FIGURE 1:**
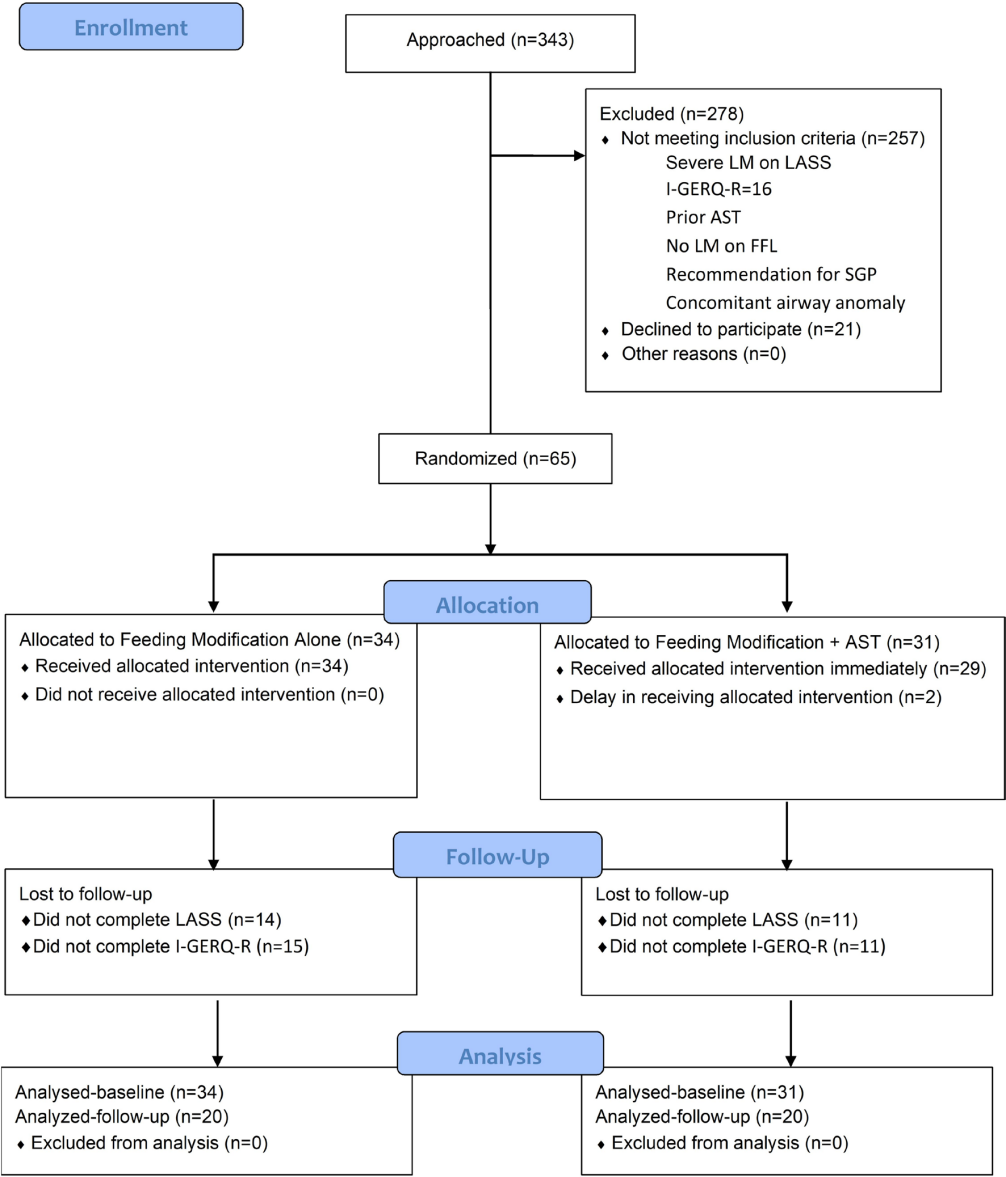
Consort diagram. [Color figure can be viewed in the online issue, which is available at www.laryngoscope.com]

Of 343 patients approached, 257 were excluded due to severe laryngomalacia on LASS, severe GER (I‐GERQ‐*R* ≥ 16), prior AST, no laryngomalacia on laryngoscopy, recommendation for SGP, and/or other airway anomaly (Figure 1). Specifically, 120 had I‐GERQ‐R scores ≥ 16 (median 19.5, range 16–32). Twenty‐one declined participation. The remaining 65 patients were randomized to AST with feeding modifications (*n* = 31, 19 moderate LASS) or feeding modification only (*n* = 34, 23 moderate LASS). Baseline characteristics were comparable between AST and no‐AST groups, including age at enrollment (AST: 2.3 months, range 1 week‐6.2 months vs. no‐AST 2.3 months, range 2 weeks‐6.8 months, *p* = 0.6), sex (AST: 14/31, 45% female vs. no‐AST: 11/34, 32% female, *p* = 0.3), and weight (AST: mean 5.8 ± SD 1.5 kg vs. no‐AST: mean 5.6 ± SD 1.3 kg, *p* = 0.6), LASS (AST: mean 2.3 ± SD 1.0 vs. no‐AST: mean 2.8 ± SD 1.1, *p* = 0.1) and I‐GERQ‐R scores (AST: median 10, range 5–15 vs. no‐AST: median 2.3, range 0.5–6.8, *p* = 0.6) at enrollment (Table 1). Private insurance was more common in the AST group (AST: 21/31, 68% vs. no‐AST: 15/34, 44%, *p* = 0.06), but the difference was not statistically significant.

**TABLE 1:** Baseline characteristics.

	Overall (*n* = 65)	No‐AST (*n* = 34)	AST (*n* = 31)	*p*
	*n* (%)	*n* (%)	*n* (%)	
Female	25/65 (38%)	11/34 (32%)	14/31 (45%)	0.3
Race				
White	52/65 (80%)	28/34 (82%)	24/31 (77%)	
Black	8/65 (12%)	3/34 (9%)	5/31 (16%)	0.5
American Indian	2/65 (3%)	2/34 (6%)	0/31 (0%)	
Unspecified	3 (5%)	1/34 (3%)	2/34 (6%)	
Ethnicity				
Hispanic or Latino	1/65 (2%)	1/34 (3%)	0/31 (0%)	
Non‐Hispanic or Latino	60/65 (92%)	31/34 (91%)	29/31 (94%)	
Unspecified	4/65 (6%)	2/34 (6%)	2/31 (6%)	
Private insurance	36/65 (55%)	15/34 (44%)	21/31 (68%)	0.06
FFL Type				
Type 1‐anterior/medial collapse of supra‐arytenoid mucosa	56/65 (86%)	29/34 (85%)	27/31 (87%)	1.0
Type 2‐short AE folds	43/65 (66%)	24/34 (71%)	19/31 (61%)	0.4
Type 3‐posterior collapse of epiglottis	8/65 (12%)	4/34 (12%)	4/31 (13%)	1.0
Pre‐LASS group				0.6
Mild	23/65 (35%)	11/34 (32%)	12/31 (39%)	
Moderate	42/65 (65%)	23/34 (68%)	19/31 (61%)	
Presenting symptoms				
Noisy breathing	59/65 (91%)	31/34 (91%)	28/31 (90%)	1.0
Stridor	57/65 (88%)	31/34 (91%)	26/31 (84%)	0.5
Emesis	34/65 (52%)	18/34 (53%)	16/31 (52%)	0.9
Choking	25/65 (38%)	15/34 (44%)	10/31 (32%)	0.3
Coughing	24/65 (37%)	14/34 (42%)	10/31 (33%)	0.5
Chest wall retractions	10/65 (15%)	8/34 (24%)	2/31 (6%)	0.09
Gagging	9/65 (14%)	5/34 (15%)	4/31 (13%)	1.0
Apnea	2/65 (3%)	2/34 (6%)	0/31 (0%)	0.5
Cyanosis	0/65 (0%)	0/34 (0%)	0/31 (0%)	
Increased respiratory rate	0/65 (0%)	0/34 (0%)	0/31 (0%)	
Follow‐up surveys complete	40/65 (62%)	20/34 (59%)	20/31 (65%)	0.6
Follow‐up weight within 1 year	47/65 (72%)	27/34 (79%)	20/31 (65%)	0.2
Follow‐up clinic visit within 1 year	36/65 (55%)	15/34 (44%)	21/31 (68%)	0.06
Supraglottoplasty	3/65 (5%)	2/34 (6%)	1/31 (3%)	1.0

	Mean (SD)	Mean (SD)	Mean (SD)	*p*
Pre‐LASS score	2.6 (1.1)	2.8 (1.1)	2.5 (1.0)	0.2
Pre‐weight	5.7 (1.4)	5.6 (1.3)	5.8 (1.5)	0.6
Initial to follow‐up survey (months)	3.1 (1.4)	3.5 (1.4)	2.7 (1.3)	0.8

	Median (range)	Median (range)	Median (range)	*p*
Pre‐I‐GERQ‐R score	10 (1–15)	11.5 (1–15)	10 (5–15)	0.5
Age at consult (months)	2.3 (0.2–6.8)	2.3 (0.5–6.8)	2.3 (0.2–6.2)	0.6

Abbreviations: I‐GERQ‐R, Infant Gastroesophageal Reflux Questionnaire‐Revised; LASS, Laryngomalacia Airway Symptom Score.

### 3.2 Treatment

No patients in the feeding modification alone group received AST at the time of their appointment. Six of 34 (18%) received a prescription later (within 1 year of initial consult). In the AST group, all participants received famotidine. Two did not receive a prescription immediately, but it was sent to the pharmacy within 1 week. The target dose for 30/31 participants in the AST group was 0.5 mg/kg, while dosing was 0.25 mg/kg for one participant. Two patients in the AST group later received lansoprazole.

Speech‐language pathologist consultation was completed for 55/65 (85%) participants. Recommendations included modified barium swallow (MBS) in 7 of these consults. Eleven of 65 (17%) had MBS completed within 1 year of the initial consult. Ten were diagnostic, with six including abnormal findings with thins: silent aspiration (*n* = 1), silent aspiration and penetration (*n* = 1), overt aspiration (*n* = 1), and penetration (*n* = 3). Thickened liquids were recommended based on MBS for three participants.

### 3.3 Follow‐Up LASS

**FIGURE 2:**
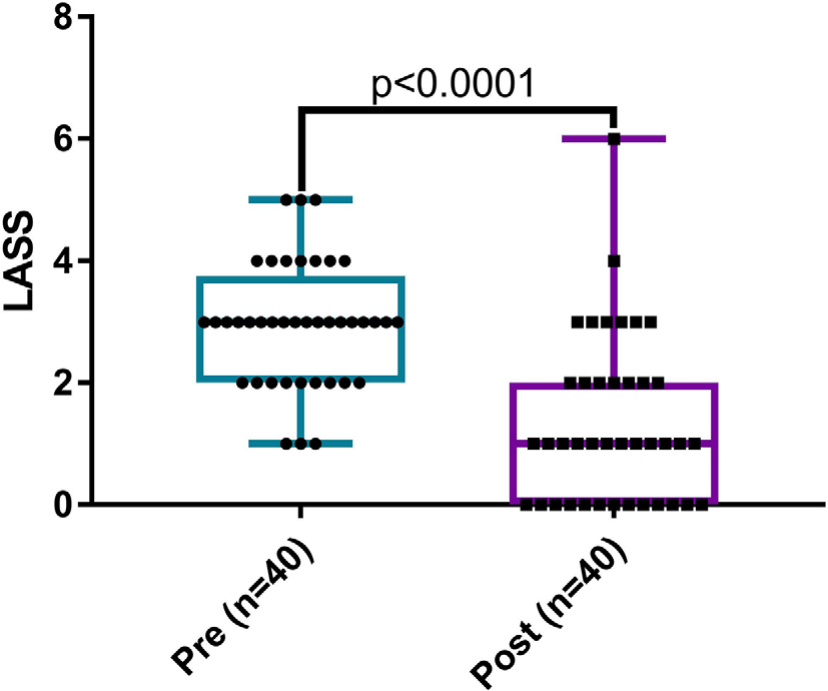
Laryngomalacia airway symptom scores (LASS) at initial visit (pre) and 3‐month follow‐up (post). There was significant improvement in LASS (Wilcoxon signed‐rank test). [Color figure can be viewed in the online issue, which is available at www.laryngoscope.com]

**FIGURE 3:**
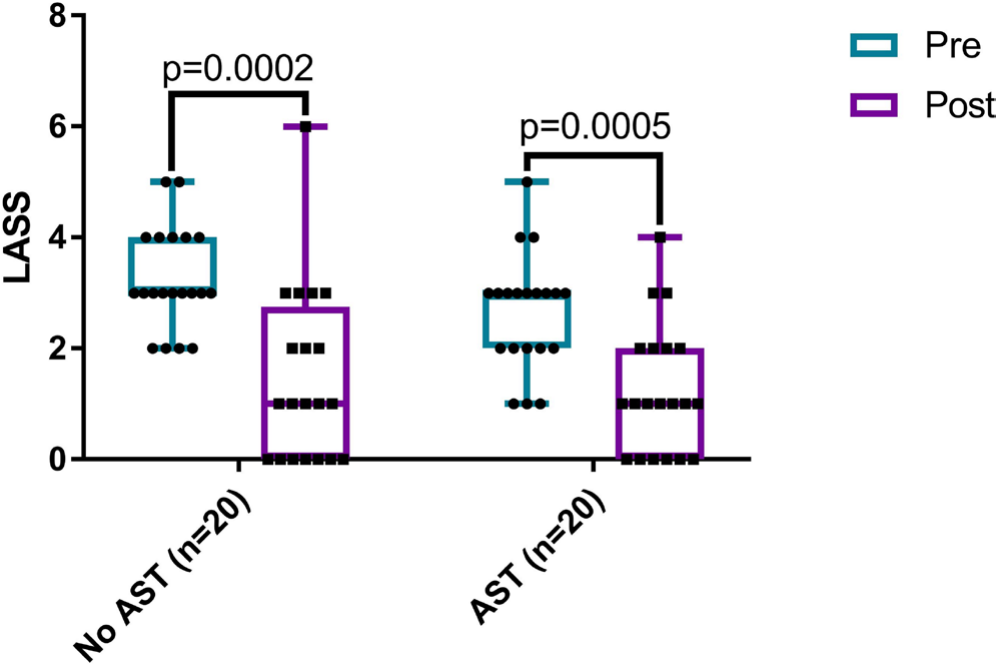
Laryngomalacia airway symptom scores (LASS) at initial visit (pre) and 3‐month follow‐up (post) by treatment group. There was significant improvement in LASS in acid suppression therapy (AST) and no AST groups (Wilcoxon signed‐rank test). [Color figure can be viewed in the online issue, which is available at www.laryngoscope.com]

Follow‐up LASS was completed by 20/31 (65%) of the AST group and 20/34 (59%) in the no‐AST group at a mean of 3.2 months (SD ±1.4 months) following enrollment. In the 40 patients with complete LASS at follow‐up, median LASS was 3 (range 1–5) at the initial appointment and 1 (range 0–6) at follow‐up (*p* < 0.0001) (Figure 2). A significant proportion (13/40; 33%) of patients exhibited resolution of laryngomalacia at follow‐up based on LASS (*p* = 0.0002) (Figure S2). Regarding the randomized group, LASS scores were significantly less at follow‐up compared with the initial visit in both patients allocated to AST (pre: median 3, range 1–5 vs. post: median 1, range 0–4, *p* = 0.0005) and feeding modification alone (pre: median 3, range 2–5 vs. post: median 1, range 0–6, *p* = 0.0002) (Figure 3), and AST did not have a significant impact on the magnitude of change in LASS scores (AST: median 1, range −1 to 4 vs. no‐AST: median 2, range −2 to 5, *p* = 0.3).

### 3.4 Follow‐Up I‐GERQ‐R

**FIGURE 4:**
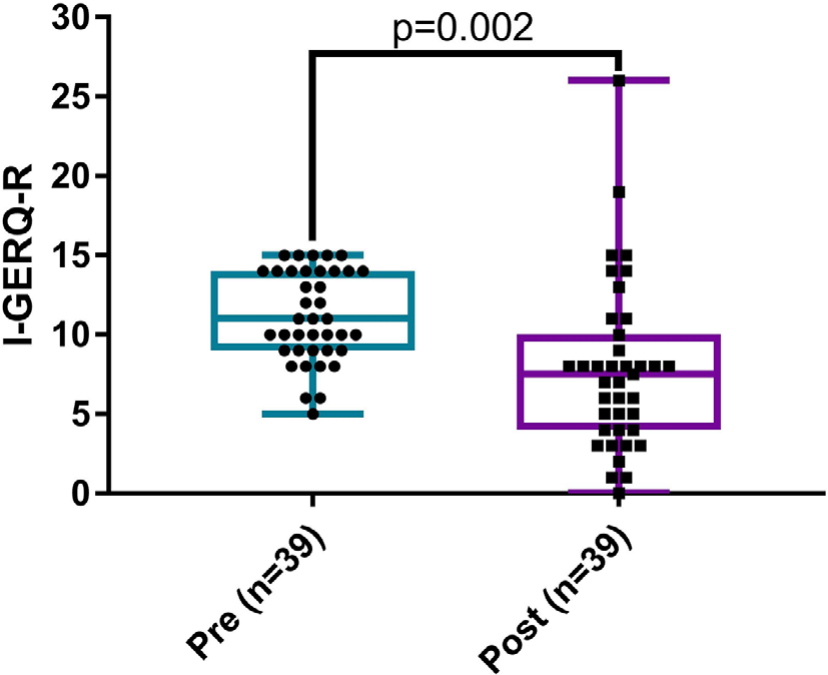
Infant Gastroesophageal Reflux Questionnaire (I‐GERQ‐R) scores at initial visit (pre) and 3‐month follow‐up (post). There was significant improvement in I‐GERQ‐R (Wilcoxon signed‐rank test). [Color figure can be viewed in the online issue, which is available at www.laryngoscope.com]

**FIGURE 5:**
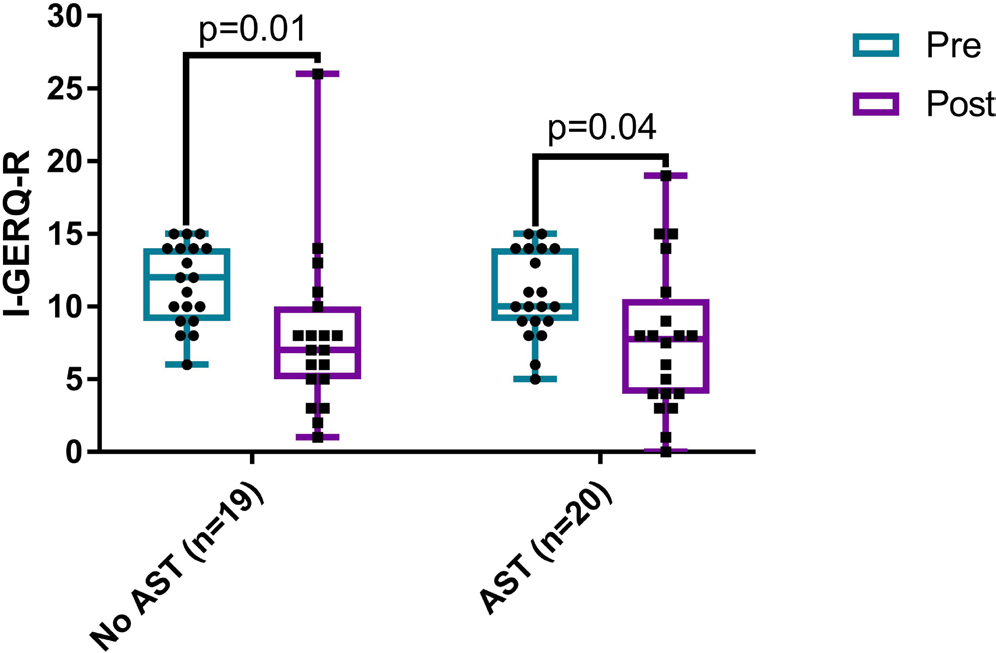
Infant Gastroesophageal Reflux Questionnaire (I‐GERQ‐R) scores at initial visit (pre) and 3‐month follow‐up (post) by treatment group. There was significant improvement in I‐GERQ‐R in acid suppression therapy (AST) and no AST groups (Wilcoxon signed‐rank test). [Color figure can be viewed in the online issue, which is available at www.laryngoscope.com]

**FIGURE 6:**
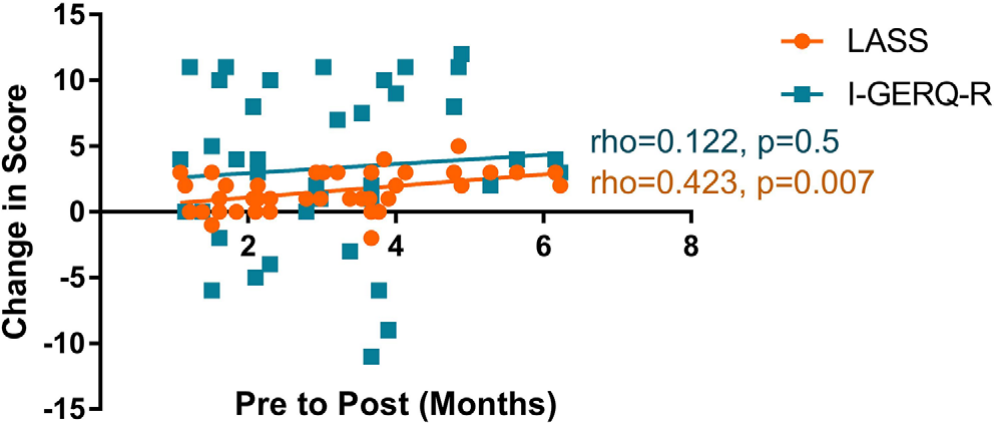
Association between change in laryngomalacia airway symptom scores (LASS) and infant Gastroesophageal Reflux Questionnaire (I‐GERQ‐R) scores and time between initial and follow‐up surveys. Improvement in LASS was positively correlated with follow‐up duration (Spearman rank correlation). [Color figure can be viewed in the online issue, which is available at www.laryngoscope.com]

Follow‐up I‐GERQ‐R was completed by 20/31 (65%) of the AST group. In the no‐AST group, follow‐up surveys were completed by 20/34 (59%), but one participant only completed questions 1–6 of the I‐GERQ‐R. In the total 39 patients with complete I‐GERQ‐R at follow‐up, median I‐GERQ‐R was 11 (range 5–15) at the initial appointment and 7.5 (range 0–26) at follow‐up (*p* = 0.002) (Figure 4). With regard to laryngomalacia severity, I‐GERQ‐R was significantly improved at follow‐up in the group with moderate laryngomalacia based on pre‐intervention LASS (pre: median 12, range 6–15 vs. post: median 7.5, range 0–26, *p* = 0.005), but not in those with mild laryngomalacia (pre: median 10, range 5–14 vs. post: median 7.5, range 1–14, *p* = 0.2) (Figure S3). However, when change scores were examined, severity of laryngomalacia at initial consult did not significantly impact the magnitude of I‐GERQ‐R improvement (moderate: median 4, range −11 to 11 vs. mild: median 2.5, range −5 to 12, *p* = 0.7). With regard to randomized group, I‐GERQ‐R scores were significantly less at follow‐up compared with initial visit in both patients allocated to AST (pre: median 10, range 5–15 vs. post: median 7.75, range 0–19, *p* = 0.04) and feeding modification alone (pre: median 12, range 6–15 vs. post: median 7, range 1–26, *p* = 0.01) (Figure 5), and AST did not have a significant impact on the magnitude of change in I‐GERQ‐R scores (AST: median 3, range −9 to 11 vs. no‐AST: median 4, range −11 to 12, *p* = 0.8). LASS (ρ = 0.423, *p* = 0.007) but not I‐GERQ‐R (ρ = 0.122, *p* = 0.5) improved more with longer time from consult to follow‐up (Figure 6).

### 3.5 Follow‐Up Weight, Symptoms, and Surgical Intervention

Weight gain over the first year of life was not significantly different between AST and feeding modification alone groups (AST: median 0.56, range 0.24–0.84 kg/month vs. no‐AST: median 0.62, range 0.27–1.04 kg/month, *p* = 0.3) (Figure S4) or between patients with mild and moderate laryngomalacia based on initial LASS (moderate: median 0.58, range 0.25–1.04 kg/month vs. mild: median 0.53, range 0.24–0.80 kg/month, *p* = 0.4) (Figure S5). When symptoms reported in clinic notes were reviewed in all 36 participants with a follow‐up clinic visit within 1 year, there was a significant decrease in odds of noisy breathing (OR: 0.083, 95% CI: 0.002–0.563), stridor (OR: 0.083, 95% CI: 0.002–0.563), and emesis (OR: 0.250, 95% CI: 0.045–0.926) at follow‐up (Table S1). When groups were analyzed separately, the improvements in noisy breathing (OR: 0.100, 95% CI: 0.002–0.703) and stridor (OR: 0.000, 95% CI: 0.000–0.694) were significant in the AST group but not the no‐AST group, while improvements in emesis were not significant in either group when analyzed separately. The odds of coughing were significantly reduced at follow‐up only in the no‐AST group (OR: 0.000, 95% CI: 0.000–0.694) (Table S1). With regard to laryngomalacia severity, the improvement in noisy breathing (OR: 0.000, 95% CI: 0.000–0.507) was significant in those with moderate laryngomalacia at the initial visit but not in those with mild laryngomalacia, while improvements in stridor and emesis were not significant in either group when analyzed separately (Table S2). SGP was performed in 3/65 (5%) participants (1 in the AST group 2.2 months following enrollment and 2 in the no‐AST group 1.4 and 1.5 months following enrollment) (Table 1). Two had moderate laryngomalacia at the initial visit and 1 had mild laryngomalacia.

## 4 Discussion

No criterion standard currently exists for the treatment of mild to moderate laryngomalacia in infants, with an absence of definitive literature comparing AST with conservative measures for the treatment of laryngomalacia‐related airway symptoms. In the present randomized, controlled trial, both airway and GER symptoms improved at follow‐up compared with the initial visit as measured using the LASS and I‐GERQ‐R, respectively. However, AST did not have a significant impact on the magnitude of improvement in LASS or I‐GERQ‐R scores. Similarly, AST did not have a significant impact on weight gain over the first year of life. These results agree with those of Sultana et al., who also determined that patients who were randomized to the AST group did not have significant changes in their I‐GERQ‐R scores or pH‐impedance metrics [16].

Notably, the only outcome that was different between AST and no‐AST groups in the current study was noisy breathing and stridor reported in clinic notes; for both symptoms, the percentage of patients with resolution was significant for the AST group but not in the no‐AST group. However, systematically collected, validated survey measures did not confirm greater improvement in GER or airway symptoms in those treated with AST. These findings, in conjunction with prior literature, suggest that patients with mild or moderate laryngomalacia and limited GER symptoms may not benefit from AST. Most laryngomalacia symptoms resolve without surgery by about 20 months of age, and starting AST early in these infants can result in significant negative sequelae. For example, studies have suggested an association between AST and increased risk of hospitalization and increased length of stay in the hospital with respiratory symptoms [11, 17]. Additionally, Duncan et al. study revealed that acid suppression use was significantly associated with a faster progression to SGP [11].

Strengths of this study include random allocation stratified by laryngomalacia severity, reducing potential bias in group allocation. The 3‐month follow‐up was longer than studies utilizing similar measures [9, 18]. In addition, the multidisciplinary airway clinic including otolaryngology and speech‐language pathology was an optimal environment for recruitment.

This study was closed to enrollment prior to reaching the target sample size due to lagging recruitment. Inclusion and exclusion criteria such as age < 6 months and LASS and I‐GERQ‐R cutoffs were imposed to ensure that participants would be comprised of the population for which the efficacy of AST therapy is in question—specifically, young infants without severe LASS or GER. However, this significantly restricted the recruitment pool. In addition, approximately 40% of patients did not complete follow‐up surveys despite multiple reminders, limiting the sample size for follow‐up comparisons and introducing potential bias related to factors influencing motivation of participants to complete follow‐up (satisfaction with treatment and/or persistent symptoms). In addition, previous literature has demonstrated the effectiveness of thickened feeds for laryngomalacia and general airway symptoms [11]. Despite contradictory evidence [18], education regarding feeding modifications that was provided to all participants in this study may have created a floor effect, making it difficult to detect additional benefit provided by AST.

Variability in follow‐up duration also introduced difficulty in discerning between improvements in symptoms due to developmental maturation of the airway versus interventions including AST and feeding modifications. Weight was recorded at the follow‐up closest to 1 year rather than at the time of survey collection because many patients returned surveys via REDCap or mail and had a visit at our hospital > 6 months after initial consult but not earlier. Acknowledging the natural history of laryngomalacia and GER includes significant improvement by 1 year of age, future studies examining the impact of AST on weight gain should obtain this data prospectively to standardize time points. Future studies should also assess compliance with AST treatment, which was not obtained in the present study. Other limitations are inherent to a single‐center study. This study was performed in a tertiary children's hospital in Western Pennsylvania. Generalizability of our findings to other regions may be limited. Multi‐institutional studies are required to adequately power and enhance generalizability of similar investigations in the future. Finally, this study intentionally excluded infants with severe laryngomalacia and those with high I‐GERQ‐R scores. Infants with more severe airway obstruction often proceed to surgical intervention, resulting in treatment heterogeneity that would confound the effects of AST. Similarly, withholding AST from patients with high reflux burden was considered unethical. As such, the findings of this study should not be extrapolated to infants with severe laryngomalacia or substantial GER symptoms, who may respond differently to AST. Future studies should examine the role of AST in these higher‐risk populations.

## 5 Conclusion

In this randomized controlled trial, we could not demonstrate additional benefit of AST over feeding modifications alone for infants with mild to moderate laryngomalacia without substantial GER, based on airway and reflux symptom scores. Given the study's limited power and high attrition, these findings should be interpreted with caution. Clinicians should carefully weigh the side effect profile of ASTs and individual patient characteristics when considering treatment options. Future multi‐institutional studies are warranted to validate and expand upon these findings.

## Conflicts of Interest

The authors declare no conflicts of interest.

## Supporting information


**Table S1:** Symptom resolution by treatment group.

**Table S2:** Symptom resolution by laryngomalacia severity.

**Figure S1:** Laryngomalacia Airway Symptom Survey.

**Figure S2:** Laryngomalacia severity based on laryngomalacia airway symptom scores (LASS) at initial visit (pre) and 3‐month follow‐up (post). There was significant resolution of LASS at follow‐up (McNemar test). Error bars indicate 95% confidence interval for proportions.

**Figure S3:** Infant Gastroesophageal Reflux Questionnaire (I‐GERQ‐R) scores at initial visit (pre) and 3‐month follow‐up (post) by initial Laryngomalacia Airway Symptom Score (LASS) severity. There was improvement in I‐GERQ‐R in those with moderate laryngomalacia (Wilcoxon signed‐rank test).

**Figure S4:** Change in weight by treatment group. There was no difference in the rate of weight gain between acid suppression therapy (AST) and no AST groups (Wilcoxon signed‐rank test).

**Figure S5:** Change in weight by initial Laryngomalacia Airway Symptom Score (LASS) severity. There was no difference in the rate of weight gain between those with mild or moderate laryngomalacia (Wilcoxon signed‐rank test).

## Data Availability

The data that support the findings of this study are available on request from the corresponding author. The data are not publicly available due to privacy or ethical restrictions.
