# Supplementary material for: Acid Suppression in Mild‐Moderate Laryngomalacia Without GERD: A Randomized Controlled Trial
Source: Laryngoscope. 2025 Aug 5;136(1):471–8. doi: 10.1002/lary.32471 (PMC12770870; doi:10.1002/lary.32471)
Supplement: Supplementary file 1 — Table S1: Symptom resolution by treatment group. [file LARY-136-471-s002.docx]

Supplemental Table 1. Symptom resolution by treatment group

|  | Overall (n=36) | | | No-AST (n=15) | | | AST (n=21) | | |
| --- | --- | --- | --- | --- | --- | --- | --- | --- | --- |
|  | Initial | Follow-Up |  | Initial | Follow-Up |  | Initial | Follow-Up |  |
|  | n (%) | n (%) | p | n (%) | n (%) | p | n (%) | n (%) | P |
| Presenting Symptoms |  |  |  |  |  |  |  |  |  |
| Noisy Breathing | 32/36 (89%) | 21/36 (58%) | **0.003** | 13/15 (87%) | 11/15 (73%) | 0.5 | 19/21 (90%) | 10/21 (78%) | **0.01** |
| Stridor | 30/36 (83%) | 19/36 (53%) | **0.003** | 13/15 (87%) | 9/15 (60%) | 0.2 | 17/21 (81%) | 10/21 (48%) | **0.02** |
| Emesis | 23/36 (64%) | 14/36 (39%) | **0.04** | 10/15 (67%) | 7/15 (47%) | 0.5 | 13/21 (62%) | 7/21 (33%) | 0.07 |
| Choking | 13/36 (36%) | 8/36 (22%) | 0.2 | 6/15 (40%) | 1/15 (7%) | 0.06 | 7/21 (33%) | 7/21 (33%) | 1.0 |
| Coughing | 15/36 (42%) | 8/36 (22%) | 0.1 | 8/15 (53%) | 1/15 (7%) | **0.02** | 7/21 (33%) | 7/21 (33%) | 1.0 |
| Chest Wall Retractions | 4/36 (11%) | 2/36 (6%) | 0.6 | 3/15 (20%) | 1/15 (7%) | 0.5 | 1/21 (5%) | 1/21 (5%) | 1.0 |
| Gagging | 4/36 (11%) | 2/36 (6%) | 0.7 | 2/15 (13%) | 0/15 (0%) | NA | 2/21 (10%) | 2/21 (10%) | 1.0 |
| Apnea | 1/36 (3%) | 2/36 (6%) | 1.0 | 1/15 (7%) | 1/15 (7%) | 1.0 | 0/21 (0%) | 1/21 (5%) | NA |

Abbreviations: NA, Not Applicable

Bold indicates p<0.05
