# Supplementary material for: Acid Suppression in Mild‐Moderate Laryngomalacia Without GERD: A Randomized Controlled Trial
Source: Laryngoscope. 2025 Aug 5;136(1):471–8. doi: 10.1002/lary.32471 (PMC12770870; doi:10.1002/lary.32471)
Supplement: Supplementary file 2 — Table S2: Symptom resolution by laryngomalacia severity. [file LARY-136-471-s004.docx]

Supplemental Table 2. Symptom resolution by laryngomalacia severity

|  | Mild | | | Moderate | | | |
| --- | --- | --- | --- | --- | --- | --- | --- |
|  | Initial | Follow-Up |  | | Initial | Follow-Up |  |
|  | n (%) | n (%) | p | | n (%) | n (%) | p |
| Presenting Symptoms |  |  |  | |  |  |  |
| Noisy Breathing | 10/12 (83%) | 8/12 (67%) | 0.6 | | 22/24 (92%) | 13/24 (54%) | **0.004** |
| Stridor | 9/12 (75%) | 4/12 (33%) | 0.06 | | 21/24 (88%) | 15/24 (63%) | 0.07 |
| Emesis | 7/12 (58%) | 4/12 (33%) | 0.3 | | 16/24 (67%) | 10/24 (42%) | 0.1 |
| Choking | 3/12 (25%) | 0/12 (0%) | 0.3 | | 10/24 (42%) | 8/24 (33%) | 0.7 |
| Coughing | 6/12 (50%) | 2/12 (17%) | 0.2 | | 9/24 (38%) | 6/24 (25%) | 0.5 |
| Chest Wall Retractions | 1/12 (8%) | 0/12 (0%) | 1.0 | | 3/24 (13%) | 2/24 (8%) | 1.0 |
| Gagging | 0/12 (0%) | 0/12 (0%) | NA | | 4/24 (17%) | 2/24 (8%) | 0.7 |
| Apnea | 0/12 (0%) | 0/12 (0%) | NA | | 1/24 (4%) | 2/24 (8%) | 1.0 |

Abbreviations: NA, Not Applicable

Bold indicates p<0.05
