# Supplementary material for: Acid Suppression in Mild‐Moderate Laryngomalacia Without GERD: A Randomized Controlled Trial
Source: Laryngoscope. 2025 Aug 5;136(1):471–8. doi: 10.1002/lary.32471 (PMC12770870; doi:10.1002/lary.32471)
Supplement: Supplementary file 3 — Figure S1: Laryngomalacia Airway Symptom Survey. [file LARY-136-471-s001.pdf]

Department of Otolaryngology
UPMC Children's Hospital of Pittsburgh
7th Floor Faculty Pavilion
4401 Penn Avenue
Pittsburgh, PA 15224

Laryngomalacia Airway Symptom Score (LASS)

1. Does your child have noisy breathing?
   - ☐ Yes
   - ☐ No
2. Does your child have coughing and/or choking while feeding?
   - ☐ Yes
   - ☐ No
3. Does your child have coughing and/or choking while feeding <u>with</u> regurgitation?
   - ☐ Yes
   - ☐ No
4. Does your child have gasping or pauses in breathing during sleep?
   - ☐ Yes
   - ☐ No
5. Does your child have retractions when breathing? For example, it looks like your baby is working hard to breath and you can see their muscles pull in-between their ribs.
   - ☐ Yes
   - ☐ No
6. Does your child have concerning weight loss?
   - ☐ Yes
   - ☐ No
7. Does your child have pauses in breathing > 20 seconds while sleeping?
   - ☐ Yes
   - ☐ No
8. Does your child have blue spells that require intervention?
   - ☐ Yes
   - ☐ No
9. Does your child have retractions when breathing requiring intervention?
   - ☐ Yes
   - ☐ No
10. Has your child been diagnosed with pulmonary hypertension or any lung abnormalities?
    - ☐ Yes; Describe ____
    - ☐ No
