# Supplementary figures and images for: Acid Suppression in Mild‐Moderate Laryngomalacia Without GERD: A Randomized Controlled Trial
Source: Laryngoscope. 2025 Aug 5;136(1):471–8. doi: 10.1002/lary.32471 (PMC12770870; doi:10.1002/lary.32471)

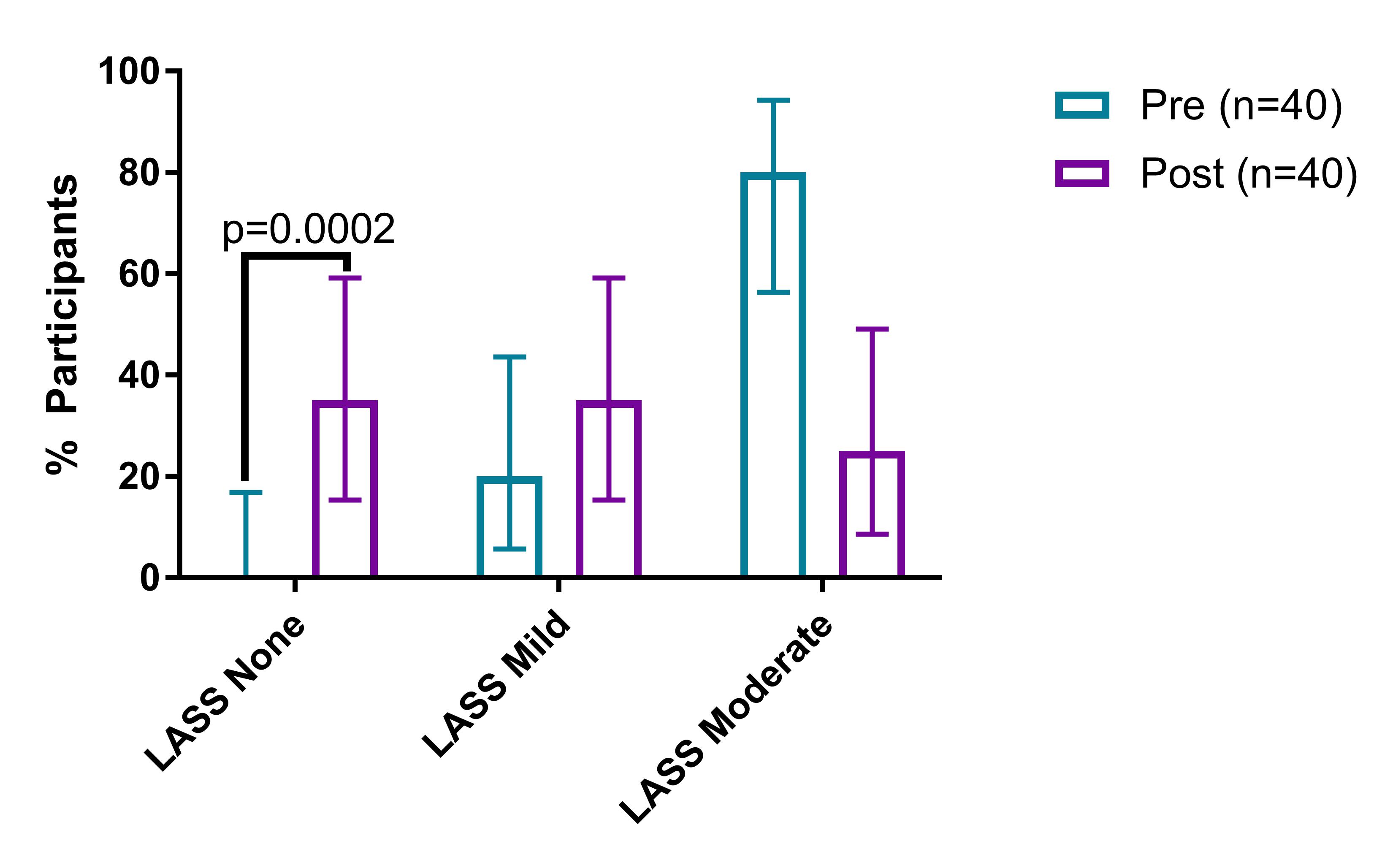

Supplement: Supplementary file 4 — Figure S2: Laryngomalacia severity based on laryngomalacia airway symptom scores (LASS) at initial visit (pre) and 3‐month follow‐up (post). There was significant resolution of LASS at follow‐up (McNemar test). Error bars indicate 95% confidence interval for proportions. [file LARY-136-471-s006.jpg]

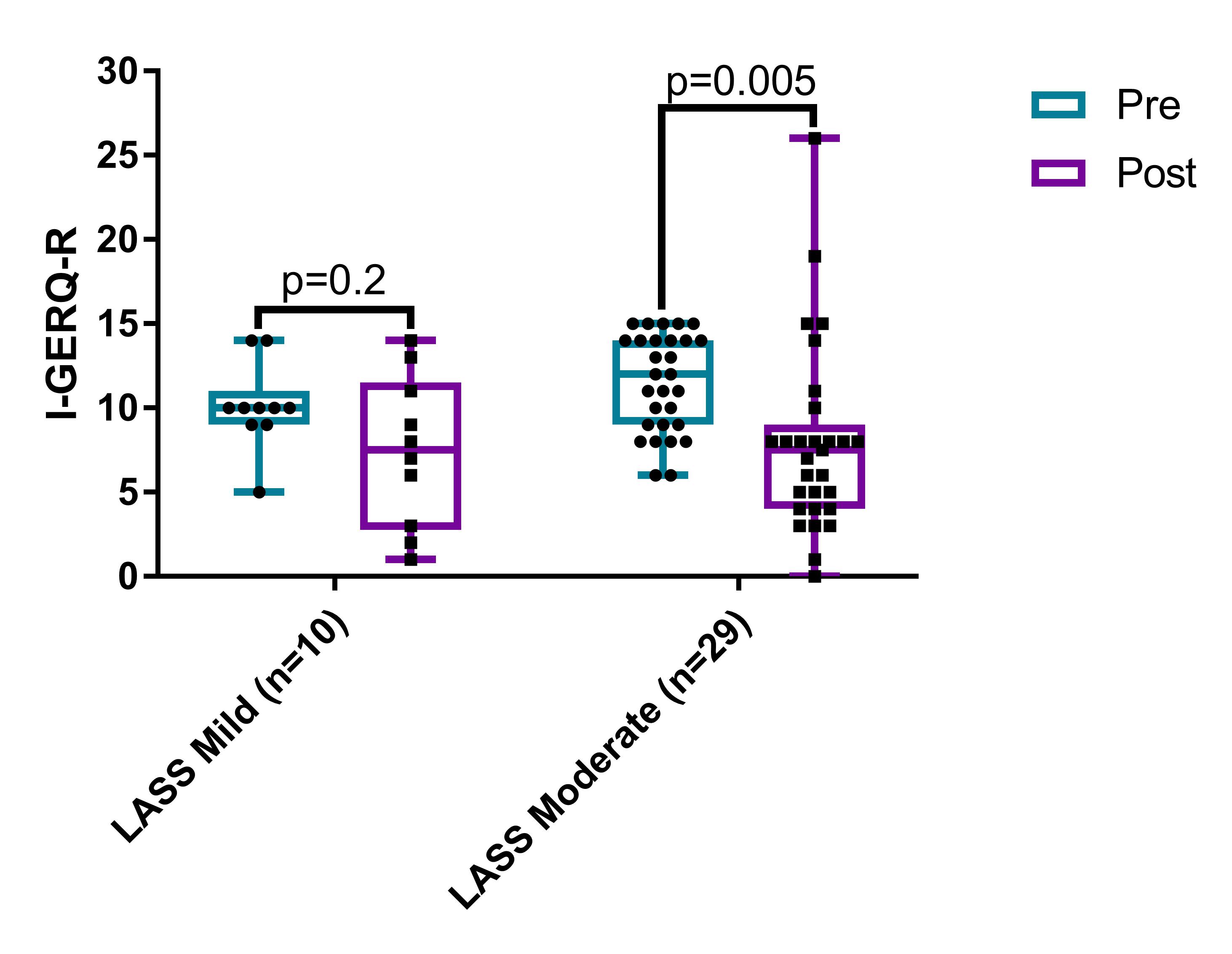

Supplement: Supplementary file 5 — Figure S3: Infant Gastroesophageal Reflux Questionnaire (I‐GERQ‐R) scores at initial visit (pre) and 3‐month follow‐up (post) by initial Laryngomalacia Airway Symptom Score (LASS) severity. There was improvement in I‐GERQ‐R in those with moderate laryngomalacia (Wilcoxon signed‐rank test). [file LARY-136-471-s003.jpg]

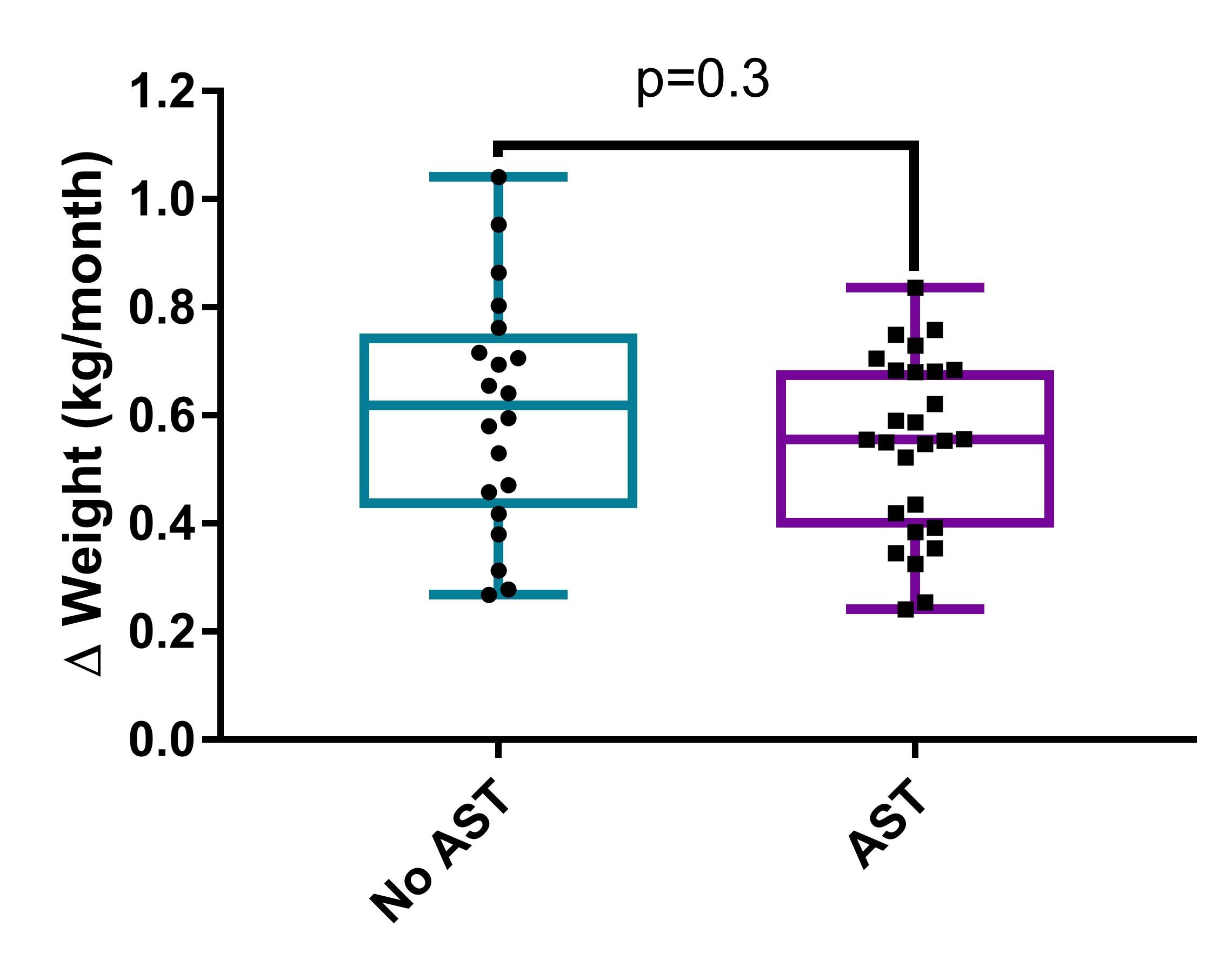

Supplement: Supplementary file 6 — Figure S4: Change in weight by treatment group. There was no difference in the rate of weight gain between acid suppression therapy (AST) and no AST groups (Wilcoxon signed‐rank test). [file LARY-136-471-s005.jpg]

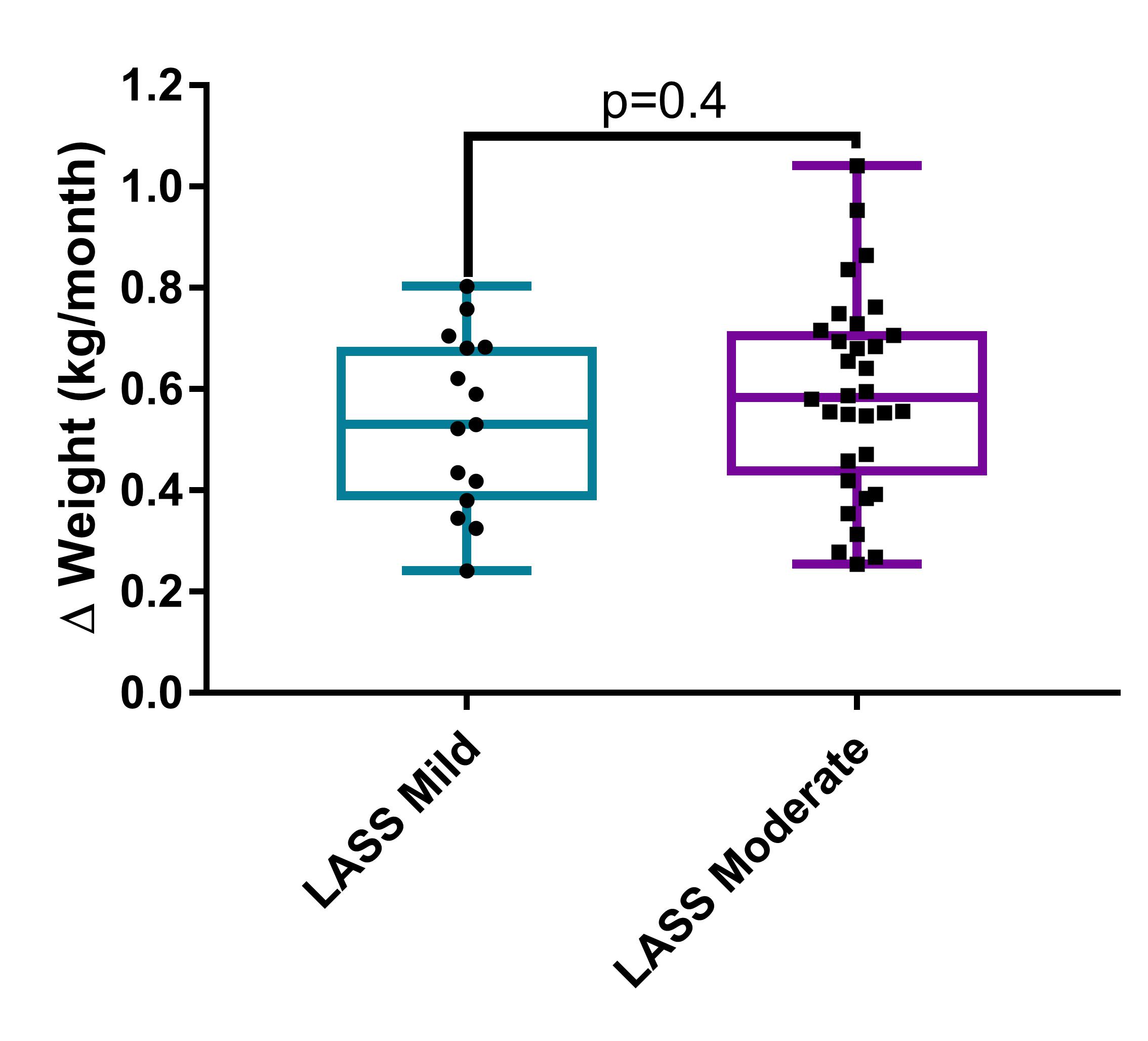

Supplement: Supplementary file 7 — Figure S5: Change in weight by initial Laryngomalacia Airway Symptom Score (LASS) severity. There was no difference in the rate of weight gain between those with mild or moderate laryngomalacia (Wilcoxon signed‐rank test). [file LARY-136-471-s007.jpg]
